# Huge fetal hepatic Hemangioma: prenatal diagnosis on ultrasound and prognosis

**DOI:** 10.1186/s12884-017-1635-7

**Published:** 2018-01-02

**Authors:** Li Jiao-ling, Geng Xiu-ping, Chen Kun-shan, He Qiu-ming, Li Xiao-fen, Yang Bo-yang, Fang Qian

**Affiliations:** 1 Department of Ultrasound, GZ Women & Children Medical Centre, Jinsui Road 9, Guangzhou, 510623 China; 2 Department of Invasive Technology, GZ Women & Children Medical Centre, Guangzhou, 510623 China; 3 Neonatal Intensive Care Unit, GZ Women & Children Medical Centre, Guangzhou, 510623 China

**Keywords:** Liver tumor, Huge hepatic hemangioma, Prenatal diagnosis, Ultrasound, Magnetic resonance imaging, Contrast-enhanced computed tomography

## Abstract

**Background:**

Although huge fetal hepatic hemangiomas are rare, they can cause fatal complications. The purpose of this study is to describe the imaging features and prognosis of these tumors.

**Methods:**

Imaging data were collected for 6 patients with huge fetal hepatic hemangiomas treated at our hospital. Imaging modalities included prenatal magnetic resonance imaging and ultrasound and postnatal color Doppler ultrasound and contrast-enhanced computed tomography (CT).

**Results:**

Among the 93,562 fetuses of 92,126 pregnant women examined at our hospital, 6 had huge hepatic hemangiomas (incidence rate, 0.64/10,000), as confirmed via postnatal color Doppler imaging and contrast-enhanced CT. Five fetuses had solitary lesions, whereas 1 (fetus 2) had multiple lesions. Four fetuses had lesions in the right liver lobe and 1 had a lesion in the left liver lobe, and 1 (fetus 2) had lesions in both lobes. All lesions showed centripetal enhancement on postnatal contrast-enhanced CT, which was more intense peripherally. Following postnatal treatment with oral propranolol, with or without dexamethasone or interventional therapy with the medical sclerosant pingyangmycin, all lesions decreased in size, with calcification plaques appearing 6 months after treatment.

**Conclusions:**

Huge hepatic hemangiomas have typical ultrasonographic features and can be diagnosed prenatally. Treatment with propranolol, with or without dexamethasone, may result in a favorable prognosis.

**Electronic supplementary material:**

The online version of this article (10.1186/s12884-017-1635-7) contains supplementary material, which is available to authorized users.

## Background

Fetal tumors are a diverse but rare group of conditions with significant implications for the health of both mother and fetus. Approximately 15% of fetal tumors are hemangiomas [1, 2], which usually appear on the face, neck and limbs. Fetal hepatic hemangiomas are benign vascular tumors present at birth and distinct from vascular malformations. They are characterized as either rapidly involuting or non-involuting based on their clinical progression [3, 4]. Fetal hepatic hemangiomas are rarely reported; hence, their exact incidence rate remains undetermined [5, 6].

Parents and clinicians are advised to regularly monitor congenital hepatic hemangiomas, which reach maximum size at birth and, in some cases, gradually disappear. Small hepatic hemangiomas are often asymptomatic and seldom require treatment. In contrast, huge hepatic hemangiomas (mean diameter > 4 cm) are less frequently reported than small hepatic hemangiomas [7] and can cause serious complications [8, 9]. Without prompt intervention, neonatal mortality rates reach 30–100% [10–12].

Hence, studies on the prenatal diagnosis of huge hepatic hemangiomas are important. Information gained by systematically monitoring fetuses for tumor progression and related complications will help obstetricians decide on timely delivery and appropriate neonatal treatments. This retrospective study examined the ultrasound characteristics and prognosis of 6 huge fetal hepatic hemangiomas in south China. To the best of our knowledge, this study is the first to report the incidence of this disease in south China and the successful use of propranolol as treatment.

## Methods

This study was approved by the ethics committee of the GZ Women & Children Medical Centre. All pregnant women in the study provided informed consent for ultrasound.

Among the 93,562 fetuses of 92,126 pregnant women examined routinely via serial ultrasound at 22–24 weeks, 30–32 weeks, and 36–38 weeks between January 1, 2013 and February 28, 2016, only 6 fetuses, of 6 women, were prenatally diagnosed with huge hepatic hemangiomas, with the diagnosis confirmed postnatally. All 6 women were referred to our center due to suspicion of fetal anomalies on ultrasound examinations at other hospitals. The mean age of the pregnant women was 27 years (range, 22–31 years).

Five of the 6 fetuses were evaluated by prenatal magnetic resonance imaging (MRI) (Signa; GE Medical Systems, Milwaukee, WI, USA), except for fetus 6, and all were evaluated by using postnatal color Doppler ultrasound and contrast-enhanced computed tomography (CT). Fetal gestational age was estimated based on the mother’s last menstrual period or via ultrasound scan performed early in the pregnancy.

A high-resolution ultrasound system equipped with a 3–5 MHz transducer (Sequoia 512; Acuson Siemens, Germany) was used to screen for fetal structural abnormalities in the middle of the second trimester. Fetuses diagnosed with hemangioma were assessed by follow-up ultrasound in our department daily, or weekly. The diagnosis of huge fetal hepatic hemangiomas was independently confirmed by 2 experienced physicians. Any complication and the mode and time of delivery were also recorded.

Neonates prenatally diagnosed with huge hepatic hemangiomas were examined immediately after birth via color Doppler ultrasound. These neonates were subsequently transferred to the neonatal intensive care unit (NICU), where they underwent contrast-enhanced CT for further evaluation. Shortly thereafter, blood concentrations of alpha-fetoprotein (AFP), neuron-specific enolase (NSE), vanillylmandelic acid (VMA), and other tumor markers were measured to rule out primary and metastatic malignant liver tumors. After performing routine blood tests and examining blood coagulation, liver and thyroid function tests, oral propranolol, with or without dexamethasone, or interventional therapy with the medical sclerosant pingyangmycin was administered [13]. The size, internal echo, and blood flow of the lesions were monitored via color Doppler ultrasound. During the neonatal follow-up period, the lesions became enlarged and surgical treatment was considered.

## Results

A total of 6 of the 93,562 fetuses of 92,126 pregnant women examined at our hospital were diagnosed with huge hepatic hemangiomas. All were incidental findings on routine ultrasound scans of the third trimester (mean, 35 + 1 weeks [range, 31+ to 39+ weeks]) at other hospitals. Five of the 6 fetuses (fetus 6 was the exception) were examined via prenatal MRI, and all were evaluated by using prenatal ultrasound and postnatal color Doppler ultrasound and contrast-enhanced CT.

**Fig. 1:**
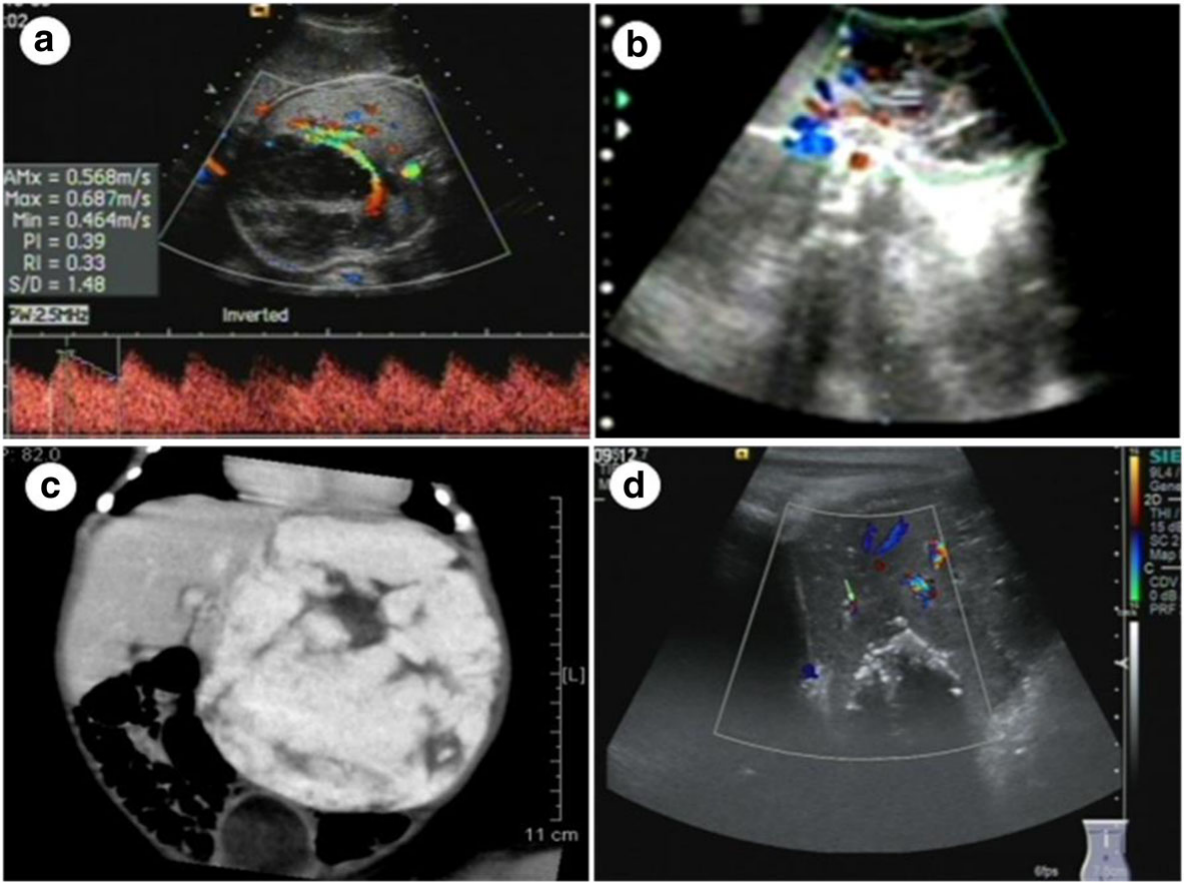
Fetus with a hemangioma in the left liver lobe (fetus 1). **a** Circular hypoechoic lesion, measuring 86 × 81 × 103 mm. Color Doppler ultrasound shows a ring of blood flow at the edge of the lesion and stars intralesion. Pulse Doppler ultrasound suggests that the resistance index is 0.33. **b** Ultrasound on the day of birth shows that lesion has a clear border, an excentric growth pattern, and an internal grid-shaped region and bypasses the left hepatic vein. **c** Postnatal computed tomography (CT) shows the lesion in the left lobe. On contrast-enhanced CT, the lesion was centripetal enhancement. **d** Two years after treatment of the neonate with propranolol and dexamethasone, the lesion was 35 × 36 × 34 mm in size, with multiple strong echo spots in the center

**Fig. 2:**
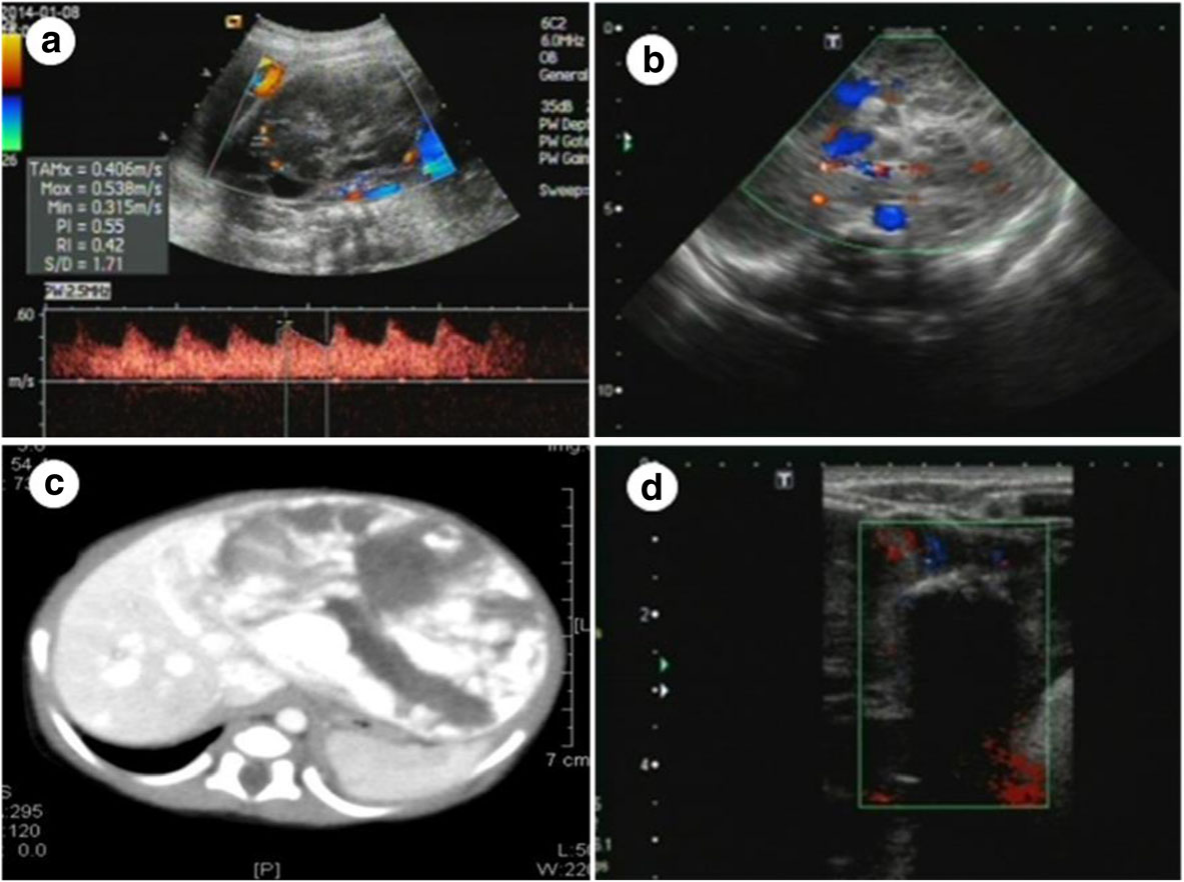
Fetus with multiple hemangiomas in the whole liver (fetus 2). **a** Largest hypoechoic lesion, measuring 92 × 56 × 80 mm and located in the left liver lobe. Color Doppler ultrasound shows a ring of blood flow at the edge of the lesion and stars intralesion. Pulse Doppler ultrasound suggests that the resistance index is 0.42. **b** Ultrasound on the day of birth shows that the large lesion has a clear border and an internal grid-shaped region. The portal vein was unobstructed, and its inner diameter was normal. **c** Postnatal computed tomography (CT) shows the large lesion in the left lobe and several smaller lesions in the right lobe. The density of the mass was low but not uniform. On contrast-enhanced CT, the edge of the lesion increased in intensity in a lattice-like pattern in the early phase of the scan, and the center of the mass gradually increased in intensity during the delay phase; there was no enhancement in the low-density zone. **d** Two years after treatment of the neonate with propranolol and dexamethasone, the lesion was 19 × 19 × 10 mm in size. Calcification plaques were observed in the lesion, and the intrahepatic and the extrahepatic bile ducts were not dilated

**Fig. 3:**
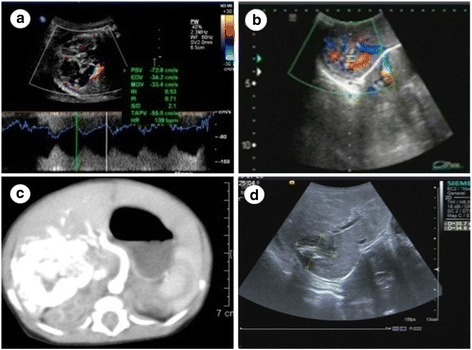
Fetus with a hemangioma in the right liver lobe (fetus 4). **a** Hypoechoic lesion measuring 56 × 42 × 41 mm. Color Doppler ultrasound shows a strip of blood flow at the edge of the lesion and stars intralesion. Pulse Doppler ultrasound suggests that the resistance index is 0.53. **b** Ultrasound on the day of birth shows that the lesion is less clear, with nonuniform density and punctate calcifications. The hepatic vein was bypassed. **c** Postnatal computed tomography (CT) shows nonuniform density and punctate calcifications. On contrast-enhanced CT, the lesion was centripetal enhancement. It was supplied by a branch of the hepatic artery and drained via the hepatic veins; the right and middle hepatic veins were thickened. **d** Six months after treatment of the neonate with propranolol and dexamethasone, the lesion was 28 × 25 × 27 mm in size. Calcification plaques were observed, and the intrahepatic and the extrahepatic bile ducts were not dilated

One fetus (fetus 2) had multiple lesions, with the other 5 having solitary lesions. Hemangiomas were present in the left liver lobe in fetus 1 and in the right liver lobe in fetuses 3–6 (Table 1). The lesions in fetus 2 varied in size, with the largest lesion in the left lobe and the smaller lesions scattered throughout the right lobe. The largest lesion in fetus 2 measured 92 × 56 × 80 mm, whereas the solitary lesions in fetus 1, 3, 4, 5, and 6 measured 86 × 81 × 103 mm, 45 × 23 × 41 mm, 56 × 42 × 41 mm, 50 × 47 × 43 mm, and 52 × 48 × 55 mm, respectively. The cardiothoracic ratio was normal in fetuses 1, 3, and 4, and above normal (<0.33) in fetuses 2, 5, and 6. The ultrasonographic features of fetuses 1, 2, and 4 are summarized in Figs. 1, 2 and 3. The boundaries of all lesions were well-defined. The internal echo was uniform in fetuses 5 and 6 and nonuniform (grid shaped) in the other 4 fetuses. The lesions in fetuses 2, 3, and 4 had a central necrotic hypoechoic area, the lesion in fetus 2 had a cystic cavity, and the lesion in fetus 4 also contained scattered punctate calcifications. Prenatal color Doppler ultrasound showed that the lesions were mainly supplied by branches of the hepatic artery and drained by 1 or 2 hepatic veins, which were usually tortuous and dilated. No abnormalities in the portal vein flow were detected on color Doppler flow images. Pulse Doppler ultrasound revealed low to medium blood flow resistance in the lesions.

**Table 1:** Ultrasonographic diagnosis and follow-up of 6 fetuses with huge hepatic hemangiomas

Fetus	GA at diagnosis (weeks)	Lesion size (mm)	Site	Cardiothoracic ratio	Delivery mode	GA at delivery (weeks)	Apgar score	Wt (g)	Sex	Postnatal therapy	Post-treatment lesion size (mm)
1	36	86 × 81 × 103	LL	Normal	CS	40	9–9-9	3120	Male	MT	35 × 36 × 34
2	31 + 6	92 × 56 × 80	BL	High	CS	37	9–9-9	3220	Male	MT	19 × 19 × 10
3	36	45 × 23 × 41	RL	Normal	CS	39 + 2	9–9-10	3220	Male	MT	31 × 33 × 26
4	34 + 2	56 × 42 × 41	RL	Normal	SD	39 + 5	9–9-9	3160	Male	MT	28 × 25 × 27
5	36 + 3	50 × 47 × 43	RL	High, TVR, little PE	SD	37 + 2	9–9-9	2710	Male	MT	20 × 20 × 17
6	37 + 1	52 × 48 × 55	RL	High, little PE	SD	38 + 3	9–9-9	2800	Male	IT	27 × 25 × 22

*Abbreviations*: *GA* Gestational age, *LL* Left liver lobe, *RL* Right liver lobe, *BL* Both liver lobes, *CS* Cesarean section, *SD* Spontaneous delivery, *MT* Medical treatment, *IT* Interventional therapy, *TVR* Tricuspid valve regurgitation, *PE* Pericardial effusion

All lesions had low signal intensity on prenatal T1-weighted MRI images and high signal intensity on T2-weighted images, relative to the signal intensity of the liver. Scattered punctate calcifications were again noted in the lesion in fetus 4. Flow voids, associated with intralesional high blood flow, were frequently seen. In addition to the large lesion in the left liver lobe in fetus 2, prenatal MRI also revealed multiple low-signal-intensity nodules of various sizes scattered throughout the right liver lobe. These nodules were spherical in shape, with clear boundaries and relatively uniform internal signal intensity. The signal intensity in the liver parenchyma between the nodules was normal. On postnatal contrast-enhanced CT, all lesions showed centripetal enhancement, which was more intense peripherally.

The obstetric information of the 6 fetuses is summarized in Table 1. Fetuses 1, 2 and 3 were delivered via cesarean section, because no signs of spontaneous labor were noted and the fetal heart rate was gradually slowing down (fetus 1), the cardiothoracic ratio had increased (fetus 2), or the uterus was scarred (fetus 3). Fetus 4 had no complications and was delivered spontaneously. Fetuses 5 and 6 were delivered spontaneously, 2 days after the occurrence of mild pericardial effusion. Postpartum Apgar scores were normal in all neonates (>8 for 1, 5, and 10 min). Neonates 1–5 received oral propranolol with or without dexamethasone, and neonate 6 underwent interventional sclerotherapy with pingyangmycin due to a relative contraindication to beta blockers. All lesions gradually decreased in size, and internal calcification plaques could be detected after 6 months. Color Doppler ultrasound showed sparse blood flow in all lesions.

## Discussion

Ultrasound is widely used in obstetrics, because it is safe for both the fetus and mother. Approximately 30% of the blood supply to the liver comes from the hepatic artery and approximately 70% from the portal vein [14], with blood drained from the liver via 3 hepatic veins. Hepatic lesions alter the pathways by which blood enters and exits the liver [15, 16], thereby aiding in the ultrasound detection of hepatic lesions.

The most likely differential diagnoses of fetal liver lesions include hepatic hemangioma, hepatoblastoma, and metastatic malignant tumor. In general, hepatic hemangiomas are well-defined mixed, solid lesions with hypervascularization and fine granular calcifications. Hepatoblastomas are spoke-wheel-like solid tumors with poor vascularization and coarse, dense calcifications. Metastatic liver tumors are solid masses with a bull’s-eye configuration and primary lesions. Malignant lesions and some benign lesions such as huge hepatic hemangiomas can cause serious complications that endanger fetal survival. The reported neonatal mortality rate of fetuses diagnosed with liver lesions is 30–100%. If huge hepatic hemangiomas are prenatally diagnosed, they can be monitored in utero to identify these potential fatal complications.

Huge hepatic hemangiomas are vascular tumors with a diameter of over 4 cm. Because these tumors are rarely reported, their actual incidence is unclear. Our study found that only 6 of the 93,562 fetuses were prenatally diagnosed with huge hepatic hemangiomas, a diagnosis that was confirmed postnatally, making the incidence of huge hepatic hemangiomas 0.64/10,000. All 6 huge hepatic hemangiomas were prenatally detected on serial ultrasounds during the third trimester at different hospitals.

Hepatic hemangiomas can occur in either or both lobes of the liver. The lesions can be solitary or multifocal and vary in size [17]. In this study, 4 fetuses had right-lobe lesions and 5 had solitary lesions. Only 1 fetus (fetus 1) had a left-lobe lesion, and only 1 (fetus 2) had multiple lesions. Cases of fetal multiple hepatic hemangiomas have only been reported twice [12, 18]. Hence, fetus 2 in this study is the third reported case in literature. The small lesions in the right lobe of fetus 2 in this study were visible only on prenatal MRI, but not on prenatal ultrasound. Postpartum color Doppler ultrasound and contrast-enhanced CT confirmed the presence of multiple small lesions in the right lobe of this fetus.

Huge hepatic hemangiomas have well-defined borderlines on 2-dimensional sonography before delivery, and are hypoechoic compared with the normal liver. Although hemangiomas have been reported hyperechoic or isoechoic, none of our lesions had such characteristics. The lesions were either uniformly or non-uniformly hypoechoic. We also observed necrotic areas with or without calcification and a cystic cavity in these lesions. Postnatal ultrasound findings were concordant with the prenatal findings in all 6 cases, and the MRI findings (described in the results section) in all 6 were in agreement with those reported by Dong et al. [19].

Both antenatal ultrasound and MRI can demonstrate larger lesions and scattered punctate calcifications, whereas prenatal MRI can reveal smaller lesions not detected on prenatal ultrasound. When prenatal ultrasound showed abundant blood flow within the lesion, flow voids associated with intralesional high blood flow were frequently observed on prenatal MRI.

Fatal complications of huge fetal hepatic hemangiomas include congestive heart failure, consumptive coagulation dysfunction, and liver rupture [18, 20]. Hence, evaluation of intrauterine conditions via serial ultrasound examinations is important in making pregnancy-related decisions, including active management with in-utero treatment, expectant management, or expediting delivery. Fetuses with suspected huge hepatic hemangiomas in our study were monitored weekly for tumor progression and complications. When the cardiothoracic ratio increased and the fetus was full term, we chose a suitable delivery method, and the infant was delivered in a timely manner. By contrast, when the cardiothoracic ratio increased prior to full term, we usually monitored the fetus daily until 32 weeks gestation, with the fetus delivered immediately upon the appearance of a pericardial effusion.

Fetuses with hepatic hemangiomas have been successfully delivered vaginally [21]. This was also true in our study, with 3 fetuses being spontaneously delivered. The mode of delivery was unrelated to lesion size, number of lesions, lesion location (right or left liver lobe) or gestational age.

Postnatally, the 6 neonates had stable vital signs. Contrast-enhanced CT, blood tests, liver and thyroid function tests, and measurements of tumor marker levels (e.g., AFP, NSE, and VMA) were performed in the NICU. There was no evidence of primary or metastatic malignant liver tumors. Multiple hepatic hemangiomas have been reported to result in hypothyroidism, but it has not been determined whether patients with rapidly involuting congenital hemangioma also had hypothyroidism. Although fetus 2 in our study had multiple lesions, his thyroid function was normal.

CT has several advantages over other imaging modalities, including a fast scanning speed, the lack of need for sedation, and low system impact, suggesting that this modality may expedite the diagnosis and treatment of neonates. All 6 neonates in this study underwent contrast-enhanced CT, which showed centripetal enhancement of all lesions, an enhancement that was more intense peripherally.

Corticosteroids have been shown to shrink hepatic hemangiomas [22, 23]. For example, multimodal management of 17 neonates included steroid treatment. One of these neonates underwent embolization as supportive medical treatment, followed by propranolol administration, but died at 1 month of age. In our study, neonates received either oral propranolol with or without dexamethasone or interventional therapy with pingyangmycin. These treatments were effective, as shown by the gradual reductions in lesion size and internal blood flow and the presence of calcification plaques 6 months after treatment.

## Conclusions

Our results suggest that accurate prenatal diagnosis and regular monitoring of fetuses with huge hepatic hemangiomas may improve prognosis and help avert serious complications. Basing postnatal treatments on prenatal imaging findings may help reduce the perinatal mortality rate due to this condition. Our findings may assist physicians in choosing the best mode and time of delivery as well as the appropriate treatment for these fetuses and neonates. To our knowledge, this study is the first to report the incidence rate (0.64/10,000) of huge congenital hepatic hemangiomas in south China. We intend to perform a prospective, controlled study to further investigate whether oral propranolol with or without dexamethasone is effective for the management of huge congenital hepatic hemangiomas.

## Additional file

Additional file 1: Description of the data and materials of 6 cases with huge hepatic hemangiomas. (XLSX 21 kb)

## Abbreviations

AFP: Alpha-fetoprotein
CT: Computed tomography
MRI: Magnetic resonance imaging
NICU: Neonatal intensive care unit
NSE: Neuron-specific enolase
VMA: Vanillylmandelic acid

## References

[1] Kamil D, Tepelmann J, Berg C, Heep A, Axt-Fliedner R, Gembruch U (2008). Spectrum and outcome of prenatally diagnosed fetal tumors. Ultrasound Obstet Gynecol.

[2] Boull C, Maguiness SM (2016). Congenital hemangiomas. Semin Cutan Med Surg.

[3] Mulliken JB, Enjolras O (2004). Congenital hemangiomas and infantile hemangioma: missing links. J Am Acad Dermatol.

[4] Cohen MM (2007). Hemangiomas: their uses and abuses. Am J Med Genet A.

[5] Dreyfus M, Baldauf JJ, Dadoun K, Becmeur F, Berrut F, Ritter J (1996). Prenatal diagnosis of hepatic hemangioma. Fetal Diagn Ther.

[6] Helmberger TK, Ros PR, Mergo PJ, Tomczak R, Reiser MF (1999). Pediatric liver neoplasms: a radiologic-pathologic correlation. Eur Radiol.

[7] Pott Bartsch EM, Paek BW, Yoshizawa J, Goldstein RB, Ferrell LD, Coakley FV (2003). Giant fetal hepatic hemangioma: case report and literature review. Fetal Diagn Ther.

[8] Chuileannain FN, Rowlands S, Sampson A (1999). Ultrasonographic appearances of fetal hepatic hemangioma. J Ultrasound Med.

[9] Aslan H, Dural O, Yildirim G, Acar DK (2013). Prenatal diagnosis of a liver cavernous hemangioma. Fetal Pediatr Pathol.

[10] Sur A, Manraj H, Lavoie PM, Lim K, Courtemanche D, Brooks P (2016). Multiple successful angioembolizations for refractory cardiac failure in a preterm with rapidly involuting congenital hemangioma. AJP Rep.

[11] Morimura Y, Fujimori K, Ishida T, Ito A, Nomura Y, Sato A (2003). Fetal hepatic hemangioma representing non-reassuring pattern in fetal heart rate monitoring. J Obstet Gynaecol Res.

[12] Franchi-Abella S, Gorincour G, Avni F, Guibaud L, Chevret L, Pariente D (2012). SFIPP-GRRIF (Société Francophone d’Imagerie Pédiatrique et Périnatale-Groupe de Recherche Radiopédiatrique en Imagerie Foetale). Hepatic haemangioma-prenatal imaging findings, complications and perinatal outcome in a case series. Pediatr Radiol.

[13] Hou J, Wang M, Tang H, Wang Y, Huang H (2011). Pingyangmycin sclerotherapy for infantile hemangiomas in oral and maxillofacial regions: an evaluation of 66 consecutive patients. Int J Oral Maxillofac Surg.

[14] Wang H (2005). editor-in-chief. Topographic anatomy.

[15] Isaacs H (2007). Fetal and neonatal hepatic tumors. J Pediatr Surg.

[16] Makin E, Davenport M (2010). Fetal and neonatal liver tumours. Early Hum Dev.

[17] Lutgendorf MA, Magann EF, Yousef M, Hill JB, Foster DT (2009). Hepatic epithelial hemangioendothelioma in pregnancy. Gynecol Obstet Investig.

[18] Gembruch U, Baschat AA, Gloeckner-Hoffmann K, Gortner L, Germer U (2002). Prenatal diagnosis and management of fetuses with liver hemangiomata. Ultrasound Obstet Gynecol.

[19] Dong SZ, Zhu M, Zhong YM, Yin MZ (2010). Use of foetal MRI in diagnosing hepatic hemangioendotheliomas: a report of four cases. Eur J Radiol.

[20] Meirowitz NB, Guzman ER, Underberg-Davis SJ, Pellegrino JE, Vintzileos AM (2000). Hepatic hemangioendothelioma: prenatal sonographic findings and evolution of the lesion. J Clin Ultrasound.

[21] Chou SY, Chiang HK, Chow PK, Wu CF, Liang SJ, Hsu CS (2005). Fetal hepatic hemangioma diagnosed prenatally with ultrasonography. Acta Obstet Gynecol Scand.

[22] Schmitz R, Heinig J, Klockenbusch W, Kiesel L, Steinhard J (2009). Antenatal diagnosis of a giant fetal hepatic hemangioma and treatment with maternal corticosteroid. Ultraschall Med.

[23] Morris J, Abbott J, Burrows P, Levine D (1999). Antenatal diagnosis of fetal hepatic hemangioma treated with maternal corticosteroids. Obstet Gynecol.

